# Parental co-residence and young adults’ mental health

**DOI:** 10.1371/journal.pone.0294248

**Published:** 2023-11-29

**Authors:** Amber Howard, Ang Li, Rebecca Bentley

**Affiliations:** 1 Centre for Health Policy, School of Population and Global Health, University of Melbourne, Melbourne, Australia; 2 Department of Human Geography, Planning and International Development, Faculty of Social and Behavioural Sciences, University of Amsterdam, Amsterdam, Netherlands; Universiti Malaysia Sarawak, MALAYSIA

## Abstract

The growing trend towards young adults staying in the parental home has garnered much recent scholarly interest. However, less is known about *which* young adults are living at home, and the impacts this has over young adults’ lives. Using The Household, Income and Labour Dynamics in Australia (HILDA) dataset, this study examines the profiles of co-residing young adults and how these have changed over the first two decades of the 21^st^ century. It then analyses the associations between co-residence and young adults’ mental health, applying a propensity score modelling approach to determine differences in mental health between young adults living at home and their counterparts living independently. Results indicate that rates of co-residence have increased over the 2000s, most steeply amongst those residing outside of major cities (by 46%), older adults (by 36%), females (by 28%), and low-income groups (by 10%). Findings show a significant negative association between co-residence and mental health (a 4-point difference on the 100-point scale, 95% CI -5.93, -2.14). However, the greatest differential in mental health between co-resident and independent young adults is observed amongst those for whom rates of co-residence have increased most dramatically, i.e., females and older adults (a 6-point difference in mental health) and residents of regional and rural areas (a 5-point difference in mental health). We situate this discussion in the context of intensifying housing market constraints, considering how the transformation of the Australian housing system into a vehicle for wealth accumulation has generated barriers to residential independence.

## Introduction

The share of young adults living in the parental home has increased in recent years after decades of decline [1, 2], with reports of rising rates across Europe [3], America [4] and Australia [5]. Whilst the proliferation of co-residence and its drivers have captured scholarly interest [6, 7], less is known about *which* young adults are living at home, and little research has been undertaken on the mental health and wellbeing consequences of this societally important change. Consequently, the role of co-residence as a determinant of mental health has not been fully explored, despite its potential contribution to increasing rates of anxiety and depression amongst younger cohorts.

Normative ages of home-leaving reflect a wide range of socio-demographic factors. Differences in rates of co-residence between countries are related to housing and labour market dynamics, education systems, welfare structures, and socio-cultural norms and expectations [8–10]. Consequently, societal changes in the timing of life course transitions, marked by prolonged years in education and later partnership formation, underpin the recent growth in co-residence rates across many countries [11]. In addition, co-residence is thought to increase following economic downturn and in times of hardship [12] and unemployment and low income have been found to reduce the probability of leaving the family home [13–15]. As such, widespread socio-economic trends towards austerity, and increased precarity in labour markets, likely play a role. In addition to these factors, housing market constraints are important drivers of co-residence: research suggests that the dissolution of available affordable rental housing has been an instrumental factor in the revival of co-residence, since in many countries rental tenancy has been the steppingstone for young adults to exit the family home [16, 17]. Supporting this, scholars in the UK [18], US [19] and Canada [11] have found that young adults were more likely to live at home in more affluent areas and regions, where housing costs are highest.

Where co-residence may once have been “a time-honoured strategy for stretching thin resources” [20], international evidence suggests the profiles of co-residing young adults are diversifying. Worth [11] describes co-residence as a way for young adults to either ‘get by’ or ‘get ahead’. For less advantaged young adults it might be the only option, whereas for other young adults co-residence acts as a resource to mitigate costs of higher education, smooth early careers characterised by (unpaid) internships and precarious entry positions, or enable saving for a down-payment [21, 22]. Co-residence therefore performs an important economic role. Its ability to “save time, money and effort”, might be particularly beneficial in urban or high-priced locations where the cost of rent and home purchase is highest, yet job and education opportunities are concentrated [11, 23].

Evidence is mixed as to what constitutes a typical co-resident household. Some studies have found that parents with co-resident children are more likely to be lower educated, lower earning and unemployed [12]. This fits with the notion that co-residence can provide “a main form of in-kind support […] especially in families who do not possess liquid assets” [24]. Other studies have highlighted the bi-directional aspect of co-residence support amongst low-income households, finding that adult children may contribute to household rent costs [25]. Other international research supports a correlation between living at home and parental resources. For example, a US study found the likelihood of staying at home increases when there is more space and the home is owner occupied [26], a German study that young adults living in single parent households left home earlier than young adults living with both parents [27], and a British study found that older siblings leave home earlier- with one possible explanation being that their exit reduces household crowding [28].

Whilst there is some international evidence concerning who lives in co-residence households, there is a paucity of knowledge of the shifting consequences of this arrangement- including in relation to young adults’ mental health and wellbeing. Some notable exceptions exist in a US context [15, 29], with literature typically drawing on theories of life course and emerging adulthood to explain associations [30, 31]. This framing describes conforming to societal norms and reaching conventional markers of adulthood as signs of achievement against which co-residing young adults fall short- impacting their mental health. Other important mechanisms in explaining the relationship between co-residence and mental health are family and housing market dynamics, yet this relationship is not self-evident. For some young adults, co-residence may offer comfort, space and resources beyond their own means [11], and in urban locations may enhance educational and labour market possibilities [18, 20]. For households with less resources, however, co-residence may bring strains and stresses, and further compound overcrowded conditions [24, 32]. However, the role of family and housing markets in shaping outcomes for co-residing young adults is less often considered in this life course framing.

To address these gaps in research, we use longitudinal data collected over 20 years on an Australian cohort to describe who lives in co-resident households and how this has changed over time. We then use a propensity score matching approach to compare the mental health of young adults with their counterparts living independently. Existing literature implies that such association is contingent on temporal factors such as economic situation, and the relatively normality of this living arrangement in each place and time [12]. We therefore draw on the most recent wave for this analysis, as to capture the contemporary association between co-residence and mental health in the current socio-economic climate. This is not an in-depth causal analysis, but rather an initial snapshot which seeks to determine if there is an association between co-residence and mental health when comparing two otherwise similar cohorts, establish the direction of this relationship, and to make a start in identifying amongst which socio-demographic groups the differential in mental health is largest. This insight into an under researched yet increasingly prevalent living situation paves the way for future work to start unpacking causality and explore the phenomena in more depth, gaining better understanding into who co-residence works well for, and who it does not.

Australia is a useful case for this enquiry in that it is emblematic of a deeply liberalised housing system. Examining co-residence in this context thus provides insight into how high house prices, low social housing availability and a lightly regulated private rental sector, against a backdrop of labour market insecurity and stringent welfare support, generate barriers to independent living that, in turn, potentially shape inequalities in mental health. Before proceeding to our analysis, we give a brief context to the Australian housing system. This provides crucial context to changes in co-residence patterns.

### The Australian case

Australia is categorised as a country with a liberal welfare regime [33], where housing wealth is actively promoted as a substitute to the post-war welfare state that emerged throughout other high-income economies [34]. Homeownership was the initial mechanism through which families were encouraged to accumulate wealth. The expansion of mortgage markets, grants and subsidies, and preferable tax incentives drove up rates of owner-occupied housing which peaked long before other high income economies- reaching 71% in 1970 [35]. However, by the turn of the century, debt driven price inflation saw the start of Australia’s “chicken and egg problem” [36], with mortgage debt and house prices rising in a self-reinforcing cycle. One outcome has been the exclusion of younger households without existing assets from home-buying possibilities.

More recently, private rental housing has become central in asset-based welfare strategies [29], with subsidies and tax benefits vigorously incentivising investment. These incentives are paired with weak private rental sector regulations which facilitate landlord profit at the expense of tenants’ rights–for example enabling frequent tenant turnovers, price increases, and low maintenance requirements. On one hand, this further drives rental housing investment, with investors- both small and large scale- providing tough competition for younger households looking to buy [34]. On the other hand, it renders private rental housing unattractive and, increasingly, unattainable for young adults. For those pushed out, there are few alternatives. A marginal social rental sector which houses around 3% of the population is not only strikingly small in scale but is heavily means tested and targeted towards those with prioritised needs [37].

Australian housing policy over the past decades has focused on responsibilising citizens to substitute a diminishing welfare state, positioning homes—both owner occupied and private rental- as vehicles for wealth accumulation and a means to independence. This has, ironically, hindered many young adults from residential independence, thus becoming increasingly reliant on their parents’ homes and resources.

## Data, variables, and methods

### Data

We use the Household, Income and Labour Dynamics in Australia (HILDA) data, a household-based panel survey carried out by the Melbourne Institute since 2001, which includes detailed information on multiple members of households. Pairing young adults to their parents, utilising detailed household identifier variables, allows us to describe both the parent and household characteristics of young adults who live at home and their counterparts who live independently.

To describe how the profiles of co-resident young adults have changed over time, we use three waves of data from 2002, 2010 and 2018. 2002 was the first year that the necessary variables for this analysis were directly comparable with later waves. Data collected in 2010 enables us to capture the period shortly after the global financial crisis. 2018 was the most recent data collected that was not impacted by the COVID-19 pandemic at the time of writing. We use data from 2018 to analyse associations between co-residence and mental health. As previously explained, we anticipate this relationship to be contingent on broader socio-economic factors, and we seek to capture associations relevant to the contemporary climate and housing market context.

### Variables

Identifier variables were used to classify parents (biological, step or foster) living with their adult children at the time that the survey was conducted. From this we derived a binary variable indicating if either or both parents were living with their adult offspring at the date the survey was conducted. Multiple alternative specifications of the exposure were tested, with the final specification selected due to the alignment of our figures with those enlisted in the official HILDA report [5]. Our comparator group of young adults living independently was restricted to adults who had a parent in the survey. The 2018 wave contained information on 5,346 young adults aged 18–34 years, 1,397 of whom resided in the parental home and 3,949 who lived independently. The sample size for 2002 was 3,633 (862 co-residents) and for 2010 was 3,945 (1,066 co-residents).

Respondents aged between 18 and 34 years were included in the sample, with 18 years of age being the legal age of adulthood and the earliest normative point of home-leaving in Australia. The upper age threshold of 34 years has been used across housing research to encapsulate ‘young adults’ [38–40]. Young adults’ and parent’s income is categorised in three quantiles interpreted as low, medium and high. Young adults’ income is computed respective to their age category, and parent income is categorised respective to the overall population. All variables, including co-residence status and socio-economic characteristics, have been independently calculated for each wave.

We use The Short Form 36 (SF-36) mental health and wellbeing scale, which is based on 36 self-completed questions about health and well-being which capture different aspects of health. Summary scores are derived for both physical and mental health, the latter of which was used in this study. Scores are measured from 0–100 on a standardised scale, with lower scores indicative of poorer health and higher score reflecting better health. Researchers emphasise that determining a minimum clinically important difference in scores is dependent on population and context. SF-36 has been recognised as a robust measure of self-rated health [41]. It has been used extensively in Australian research on the relationships between housing and health [42–44].

### Methods

All analysis has been undertaken using Stata SE 17.0. We begin by using descriptive statistics to identify who is living in co-resident households and how this has changed over time, applying population weights to increase representation in sampling. For our statistical analysis, we use propensity scoring matching to compare the mental health of young adults living at home with their socio-demographically similar counterparts living independently. We estimated the propensity for respondents to be co-resident, controlling for the covariates age, sex, education level, income, labour force status, Aboriginal and Torres Strait Islander origin, parent’s education, parent’s income, parent’s remoteness, parent’s tenure and parent’s dwelling type (S1 Table). This selection of covariates was guided by literature [12, 13]. Next, we used the psmatch2 command to match young adults living independently to young adults living at home based on their propensity score with one-to-one ratio.

We checked the quality of matching through pseudo-R2, Rubin’s B and Rubin’s R (S2 Table) and balance diagnostic tests (S1 Fig), with a commonly used measure of matching quality being standardised mean difference of <10% indicating sufficient balance. Once satisfied with the matching, we used a weighted linear regression model to estimate the difference in mental health between young adults living at home and young adults living independently, controlling for the defined covariates in a doubly robust estimation. We stratified by a range of individual, parent and housing characteristics. Sensitivity analysis undertaken through matching with different calipers (0.1 and 0.3), and through restricting analysis to households with just one young adult living at home. All demonstrated similar figures in the same direction, and therefore have not been displayed due to very close alignment with final results. Sensitivity analyses thus support our general conclusions on the association between co-residence and mental health.

## Results

The share of young adults living at home in our sample has risen by 18% between 2002 and 2018 (Table 1). Rates of co-residence are highest amongst younger adults, but the steepest increases over the time period can be seen amongst the oldest cohort. Additionally, our data highlights notable increases in co-residence amongst low income groups, amongst females, and amongst those married or living with partners. Findings in Table 1, and subsequent reported figures, should be interpreted with confidence intervals in mind.

**Table 1:** Characteristics of co-resident young adults, 2002, 2010 and 2018, % (95% confidence intervals).

Characteristics	2002		2010		2018		% difference 2002–18
	(n = 862)		(n = 1,066)		(n = 3,949)		
	%	95% CI	%	95% CI	%	95% CI	
**Total**	34	(31.72–35.89)	38	(36.32–40.64)	40	(37.32–41.81)	+18
**Age**							
*18–21 years*	72	(69.08–75.59)	78	(75.14–80.39)	80	(76.94–83.20)	+11
*22–25 years*	44	(39.06–48.08)	47	(43.24–51.05)	54	(49.45–57.84)	+23
*26–29 years*	22	(18.06–26.34)	24	(20.06–29.40)	21	(17.87–24.10)	-4
*30–34 years*	11	(8.86–14.61)	12	(8.84–16.42)	15	(11.44–20.16)	+36
**Sex**							
*Female*	29	(26.71–31.60)	34	(31.81–36.52)	37	(33.85–39.90)	+28
*Male*	38	(36.05–40.94)	43	(39.97–45.51)	42	(39.58–44.98)	+11
**Income**							
*Low income*	52	(48.09–54.99)	57	(54.02–60.43)	57	(52.76–61.31)	+10
*Middle income*	32	(28.31–36.04)	36	(32.23–39.97)	33	(28.98–36.23)	+3
*High income*	17	(13.32–20.99)	20	(15.90–24.95)	18	(14.81–22.81)	+6
**Geography**							
*Major city*	36	(33.99–37.69)	31	(27.96–33.78)	34	(31.58–37.08)	-6
*Outside of major city*	28	(24.90–31.43)	41	(39.26–43.47)	41	(39.02–43.94)	+46
**Married or living with partner**	3	(2.04–4.46)	4	(2.74–5.06)	6	(4.55–8.64)	+100

Where young adults’ incomes are negatively correlated with co-residence, in our sample parental resources are associated with increased rates of co-residence (Table 2). We find that, compared to the parents of young adults not living at home, co-resident parents are most likely to earn more, be in partnerships, and live in more expensive houses, detached houses, and in major cities. They are less likely to live in social rental housing, and in flats. Subsequent descriptive analysis for earlier years (S3 Table) shows the disparity in mean parental income and in housing characteristics between co-resident and independent young adults has increased over time.

**Table 2:** Parental socio-demographic and housing characteristics by young adults’ co-residence status, 2018, % (95% confidence intervals).

	Co-resident (n = 1,397)		Independent (n = 3,949)	
	%	95% CI	%	95% CI
**Parent(s) married or living with partner**	89	(86.69–91.12)	87	(84.36–89.04)
**Parent’s highest income (mean) $**	93,709.00	(88,841–98,579)	81,003	(76,178–85,827)
**Parent’s highest house value (mean) $**	1,011,683	(95,791–106,545)	805,916	(77,150–84,033)
**Parent’s housing cost quote (% of income)**	19	(17.21–21.32)	20	(18.61–22.10)
**Parent’s tenure**				
*Homeowner*	79	(76.40–82.20)	79	(77.18–81.82)
*Private renter*	17	(14.70–20.09)	16	(14.35–18.44)
*Social renter*	3	(2.29–4.80)	4	(2.97–5.65)
**Parent’s dwelling type**				
*Flat*	4	(2.77–5.53)	8	(5.80–10.62)
*Semi-detached*	5	(3.26–6.62)	5	(3.41–6.14)
*Detached*	91	(89.07–93.30)	87	(84.64–89.74)
**Parent’s location**				
*Major city*	77	(74.38–79.73)	55	(51.98–57.74)
*Outside of major city*	23	(20.27–25.62)	45	(42.26–48.02)

There is variation in the average mental health scores of co-resident and independent young adults across a range of socio-demographic characteristics (Table 3). Differences are notable between the youngest and the oldest groups, with better mental health amongst co-resident young adults compared to independent young adults amongst the youngest cohort, which reverses amongst the older age group. Co-resident females have worse mental health than independent females, with no difference observed between co-resident and independent males. Our data highlights worse mental health amongst co-resident young adults in the lowest income group compared to their independent peers. This did not hold amongst middle and high earners.

**Table 3:** Mental health of young adults by socio-demographic and parental housing characteristics, stratified by co-residence status, 2018, mean (standard deviation).

Young adults’ mental health	Co-resident (n = 1,397)	Independent (n = 3,949)
**Total**	68 (18)	70 (18)
**Age**		
*18–21*	69 (19)	66 (21)
*22–25*	69 (18)	71 (18)
*26–29*	69 (17)	69 (18)
*30–34*	64 (15)	72 (17)
**Sex**		
*Female*	66 (19)	70 (19)
*Male*	71 (17)	70 (17)
**Income**		
Low	68 (19)	70 (17)
Middle	71 (16)	71 (17)
*High*	73 (13)	73 (17)
**Parent’s tenure**		
*Homeowner*	69 (17)	70 (18)
*Renter—Private*	66 (20)	63 (22)
*Renter—Government*	66 (23)	67 (17)
**Parent’s dwelling type**		
*Flat*	63 (23)	70 (14)
*Semi-detached*	68 (16)	70 (17)
*Detached*	69 (18)	69 (19)
**Parent’s location**		
*Major city*	69 (17)	69 (17)
*Outside of major city*	67 (23)	69 (20)

Table 4 reports the *differences* in mental of co-residing young adults compared to their peers living independently. Findings suggest a strong negative association between co-residence and mental health, with the mental health of co-residing young adults estimated to be 4.03 points lower than their independent peers after adjusting for a range of individual, parent and housing related variables (95% CI -5.93 to -2.14). Stratification by age reveals a gradient in mental health scores. In the youngest age cohort, co-residence is positively associated with mental health (although confidence intervals cross the null). Amongst the oldest age groups, co-residence is significantly negatively associated with mental health, with a decrease of approximately 1 point in mental health between each age category. This indicates that the negative association between co-residence and mental health intensifies with age. Importantly, it also implies that if we were to restrict our sample to an older subgroup, the 4.03 differential in mental health would likely be larger.

**Table 4:** Results from regression with propensity score weighting describing associations between co-residence and mental health, by socio-demographic and parental characteristics, 2018.

Variable	Co-efficient	P-value	95% Confidence interval
**Total**	-4.03	0.000	(-5.93–-2.14)
**Age**			
*18–21*	2.48	0.151	(-0.91–5.87)
*22–25*	-3.69	0.030	(-7.02–-0.35)
*26–29*	-4.50	0.022	(-8.36–-0.64)
*30–34*	-5.65	0.003	(-9.41–-1.89)
**Sex**			
*Male*	-2.38	0.071	(-4.97–0.20)
*Female*	-6.24	0.000	(-8.89–-3.60)
**Income**			
*First tercile (lowest)*	-4.48	0.004	(-7.53–-1.44)
*Second tercile*	-2.81	0.038	(-5.48–-0.15)
*Third tercile (highest)*	-4.74	0.013	(-8.49–-1.00)
**Parent’s location**			
*Outside of major city*	-4.99	0.002	(-8.07–-1.91)
*Major city*	-2.56	0.026	(-4.82–-0.30)
**Parent’s tenure**			
*Owner*	-4.35	0.000	(-6.46–-2.24)
*Private renter*	-4.11	0.059	(-8.37–0.15)
*Social renter*	-10.10	0.089	(-28.20–-2.02)
**Parent’s dwelling type**			
*Flat*	-3.58	0.526	(-14.77–7.62)
*Semi detached*	2.80	0.300	(-2.54–8.14)
*Detached*	-4.40	0.000	(-6.40–-2.39)

There is a notable difference in the mental health of females and males, with co-residing females experiencing an estimated -6.24-point difference in mental health score (95% CI -8.89 –-1.89) compared to their independent peers, far surpassing the -2.38-point difference in mental health (95% CI -4.97–0.20) between co-residing and independent males. Amongst all income groups, mental health is worse amongst co-resident young adults than their independent peers, although the differentials are highest for low and high income co-resident young adults.

Considering the differentiated effect of co-residence on mental health across a range of parent and housing related indicators (Table 4), we find a notable differential mental health effect by location. Co-residing young adults outside of urban areas report a 5-point differential in mental health compared to their independent peers; double that reported by young adults co-residing in major cities. Additionally, young adults with parents living in social housing have particularly low mental health compared to those living independently with similar characteristics.

## Discussion

To our knowledge, this study is the first of its kind to explore associations between co-residence and mental health in Australia, adding to existing work primarily undertaken in a US context [12, 29]. Both countries have historically valued independence, but have seen amounting housing, welfare and labour market constraints that have left young adults more dependent on their family’s home and resources.

We begin by addressing our first research question, describing who lives in co-resident households and how this has changed over the 21^st^ century. Our findings indicate that, in our sample, rates of co-residence have risen by around 18% in the past two decades. However, this shift has not been even across socio-economic and demographic groups. We observe a clear age gradient in co-residence trends, with eight out of ten 18 to 21 year olds living in the parental home in 2018. This decreased incrementally with age. Overtime, however, the steepest increase in co-residence can be seen amongst the oldest age group studied, with those 30 to 34 years of age seeing a 36% increase in co-residence between 2002 and 2018. This contrasts an 11% increase amongst young adults between 18 and 21 years of age. Growth amongst the older group *in particular* perhaps correlates with rising costs of renting, which for many young adults is incompatible with saving for a downpayment. This might also explain the doubling of co-residence rates amongst young adults who are married or living with partner over the 16 year period studied.

Young adult’s likelihood of leaving home has been found to increase with their income [13–15], and our data indicates that Australia is no exception in this international trend. At 57%, the prevalence of young adults in the lowest income quintile residing with parents is far greater than the 18% reported in the highest income quintile. Conversely, co-resident parents are, on average, higher earners, and more likely to be married or living with a partner than parents with independent young adults in our sample. Their relative affluence is also reflected in their housing characteristics, with co-resident parents reporting a 20% higher average house price compared to the parents of independent young adults. Co-residence rates seems to be higher in households with more living space: the parents of co-resident young adults are more likely to live in a detached house and less likely to live in an apartment than the parents of their independent peers. Our data shows no difference in homeownership rates between the parents of co-resident and independent young adults, despite research suggesting that homeowning households may have more resources available to support their offspring [45]. No observable relationship between co-residence and tenure in the Australian case might be explained by the overall high rates of owner-occupancy across income groups in the older generations.

What do these data tell us about young adults’ changing position on the housing market? On one hand, our data points to growing polarisations in housing opportunities between young adults. Whilst socio-economic divisions have been well rehearsed in literature in terms of tenure [46, 47], our data demonstrates that less affluent young adults are not only living in the private rental sector at much greater rates and purchasing houses much less, but are increasingly restricted to the family home. Co-residence is therefore an important yet under recognized area where socio-economic and age-based housing inequalities play out, marking another way that young adults’ housing outcomes have come to reflect wider patterns of socio-economic inequalities [48]. Our findings reiterate those of other studies, which suggest that *intra-*generational housing divisions are growing alongside intergenerational ones [39, 49–51].

On the other hand, our findings describe a diversification of young adults living at home. Where the youngest and lowest-income groups might live in the parental home at the greatest rates, the changes over time amongst different groups paint a more nuanced picture of worsening affordability and barriers to housing access across the socio-demographic spectrum. Some of the steepest rises in co-residence are seen in older adults, those married or living with a partner, and those residing outside of major cities. This suggests that housing market constraints have extended past groups not traditionally the most affected: dual earning households, young adult households in their 30s who are assumed to be better positioned on the labour market, and those living rurally where competition for housing has typically not been most intense, are also impacted. This is another manifestation of the post-global financial crisis housing landscape. Across socio-demographic groups (Table 1), the most notable shifts in co-residence typically happened between the first two waves surveyed (2002 to 2010). This may imply that housing market constraints were particularly amplified following the global financial crisis but that these constraints have persisted and worsened over time.

Next, we turn our attention to our second research question concerning associations between co-residence and mental health. We find a significant 4.03-point decrease in the mental health of co-resident young adults compared to their independent peers on a 100-point scale. However, just as co-residence growth has not been even across socio-economic and demographic groups, associations with mental health are also highly differentiated. Of interest is that young adults 18 to 21 years of age report better mental health than those living independently. One explanation is that independent young adults in this age cohort rely most heavily on the bottom end of private rental housing and may be more likely to face unaffordable rents, precarity and poor quality housing [52], contributing to the relative advantage of the parental home. Amongst all older age groups, we find significant negative associations between co-residence and mental health, which intensify with age.

A correlation is seen between co-residence and income in our data. Across all income groups, mental health amongst co-residing young adults is lower than for those living independently, however the differential is higher for low- and high-income young adults compared to middle income groups. Reasons for this may be that living at home may be a necessity for the lowest income young adults, who co-reside against their will. For the highest income groups this difference might be explained by relative disadvantage, with those living at home perceiving themselves as falling short of self, parental or societal expectations compared to other high-earning peers who are more likely to live independently [9, 18]. The complexity of the relationship between co-residence and income reflects the heterogeneity of co-residers as often identified in the literature: as pointed out by Kajta and colleagues [53], this population includes ‘appreciated nesters’ who may embrace the comfort of living at home, and ‘burdensome nesters’ who face little choice but to co-reside.

Important differences were also observed by region and tenure. First, we found a 5-point difference in mental health between co-residing young adults whose parents live outside of urban areas and their independent peers. This is approximately double the differential reported by those co-residing in major cities, where educational, social and career benefits may mitigate some of the negative implications of co-residence. Second, whilst little difference is noted between the mental health of co-residing young adults whose parents are in owner-occupancy or private rental housing (which both resemble the average differential in mental health), co-residing social renters reported a 10-point difference in mental health compared to their peers living independently. Strained space and fewer resources may explain this high differential.

There are limitations to this study. The structure of the data means that parents are identified if they are or have been in the sample, meaning that it excludes persons whose parents were unable to participate in the survey (e.g., living outside of Australia). We use population weights to increase representativeness of our sample and compared our figures indicating co-residence to those generated from national level census data (Australian Bureau of Statistics). Whilst the same stratifications were not available, estimates between the ABS and HILDA were broadly aligned, giving us confidence in the computation of our co-residence variable and in our descriptive figures overall. Due to the structure of the co-residence variable which simply tells us if young adults and parents live in the same household, we cannot determine whether young adults are living with parents or parents are living with young adults. Despite this, we anticipate the latter to comprise of a small number in the Australian context, where such arrangements are not culturally typical.

Other factors may impact the association between co-residence and mental health, such as clustering of mental health within households. Analysis restricting the analytical sample to households with a single young adult (i.e., with no clustering of mental health reporting amongst young adults in the same household to account for) exhibits significant results in the same direction, supporting our findings (not shown due to similarity of figures with final results). While robust adjustment has been made for confounding, we are unable to infer causality in the relationship between co-residence and mental health. Because our analysis is cross sectional, we cannot discount that it is younger people with poorer mental health who choose or require co-residence. We note, in support of an effect of co-residence on mental health, that studies have reported an increase in depressive symptoms for young people upon moving back into the family home, even after accounting for prior depressive symptoms [15]. Accordingly, we anticipate that some of the differences in mental health that we have described in these analyses between co-resident young people and their peers is related to co-residence itself, rather than the other way around. Irrespective of the main direction of effect, our findings identify a vulnerable cohort of young adults living in the parental home- which is growing in share over time.

This study has highlighted critical areas for future research. Our initial snapshot of the data suggests there may be a significant negative association between co-residence on mental health. The next step in research is to examine this using more advanced causally-focussed methods that utilise panel data fully. Further analyses of factors such as income, household composition, migration background, and age as mediators of this relationship is needed to examine who benefits from co-resident living arrangements, and for whom it has a negative impact. In addition, longitudinal analysis would enable exploration of more nuanced variables describing patterns of co-residence. Existing literature implies that ‘boomeranging’ might be an important factor in explaining the co-residence and mental health relationship [15, 27, 29], highlighting a need to consider whether young adults have lived independently prior or resided consistently at home. Other important variables facilitated with panel data include length of co-residence, age of onset, and how co-residence intersects with other living situations: does it matter for mental health if co-residence succeeds housing affordability stress, or proceeds home-purchase? Moving beyond consideration of young adults, an important area for analysis is mental health effects of co-residence on parents and other household members, too.

## Conclusions

This study has applied robust methods to a population-based dataset which contains rich information on members of households across a range of demographic, socio-economic and health-related indicators. A particular strength of this data is the ability to consider the characteristics of co-residing young adults in relation to their parents, allowing us to examine two generations in tandem. This research has provided insight into who is living in co-resident households, and how this has changed over time. It gives an initial snapshot understanding of associations between co-residence and mental health, stratifying by individual, housing and parent characteristics. We have situated this discussion in a housing market context, considering how decades of promoting housing as a substitute for public welfare and a means of securing households’ futures has, ironically, made it increasingly difficult for the younger generation to achieve residential independence. Unsurprisingly, then, our descriptive findings show that co-residence growth has not been equal: some socio-demographic groups live at home at much greater rates than others, noting a clear economic gradient is observed.

The results of our study call into question some of the assumptions that have underwritten Australian social policy for the past decades, namely that individuals are responsible for meeting their own needs and young adults’ worsening position on the housing market can be mitigated with family resources. Increased reliance on the family as a primary source of welfare has important repercussions. As Swartz [54] states, “the private and hidden nature of family support […] discourag[es] systemic solutions to shared social problems”. This is particularly salient in the Australian case where household-based welfare strategies remain deeply incentivised, and little is done to address some of the fundamental shortcomings of the housing system. Weak private rental sector regulation, and inadequate supply of social housing, are persistent issues which put pressure on parents to assist their children in a difficult housing market. Like other forms of parent support, however, living at home is available to some but not others. A growing reliance on co-residence hinders social mobility and risks driving further wedges between young adults with resourceful and urban parents, and those without.

Following research that has explored the effects of housing on mental health in relation to tenure and affordability [43, 44, 55, 56], precarity [57], and other housing conditions [58–61], this study has taken a crucial first look at associations between co-residence and mental health in the Australian case. Our findings suggest that co-residence may be another way in which housing shapes population mental health. We report notable disparities, with some young people faring much better in their mental health than others when living at home. Interestingly, the socio-demographic groups amongst whom co-residence has increased most intensely in share are also the ones who have the poorest mental health scores whilst living at home. This study therefore calls into question the assumed suitability of the parental home as a solution to young adults’ worsening positions on the housing market. Not only is co-residence not available to everyone but, importantly, it may come with notable trade-offs in mental health for those who can access it.

## Supporting information

S1 Fig Propensity score matching and standardized percentage bias. (DOCX)

S1 Table Table of covariates used in regression. (DOCX)

S2 Table Measurements of quality in propensity score matching. (DOCX)

S3 Table Results from descriptive analysis describing parental socio-demographic and housing characteristics of young adults by co-residence status, 2002*, 2010 and 2018, %. *Data on parents of young adults was different in earlier waves because this first waves of data collection did not include information on parents not living with the child in years prior to the data collection. For this reason, data from this wave has not been used to compare changes in prevalence over time.
(DOCX)
