# Supplementary figures and images for: Parental co-residence and young adults’ mental health
Source: PLoS One. 2023 Nov 29;18(11):e0294248. doi: 10.1371/journal.pone.0294248 (PMC10686488; doi:10.1371/journal.pone.0294248)

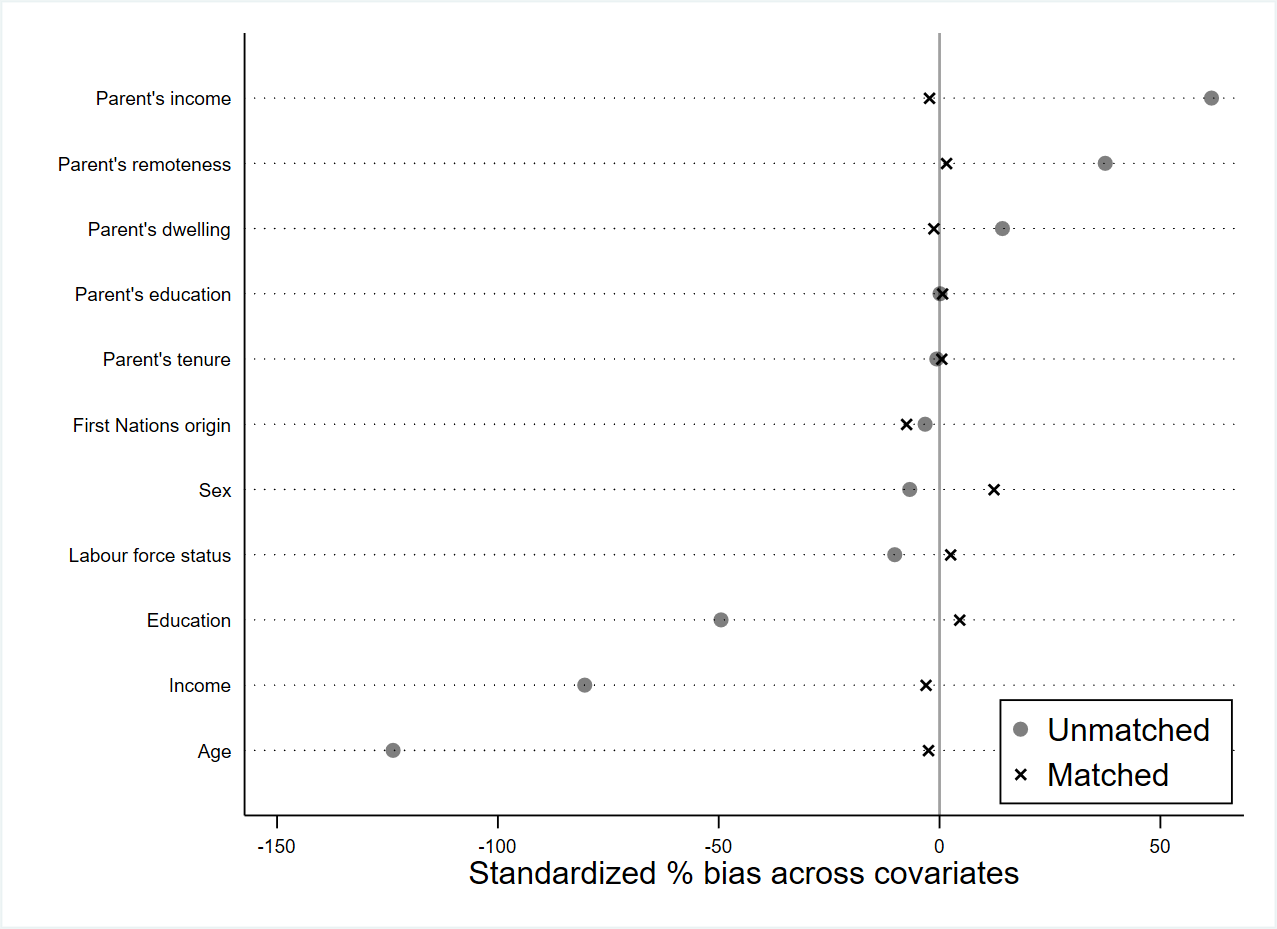


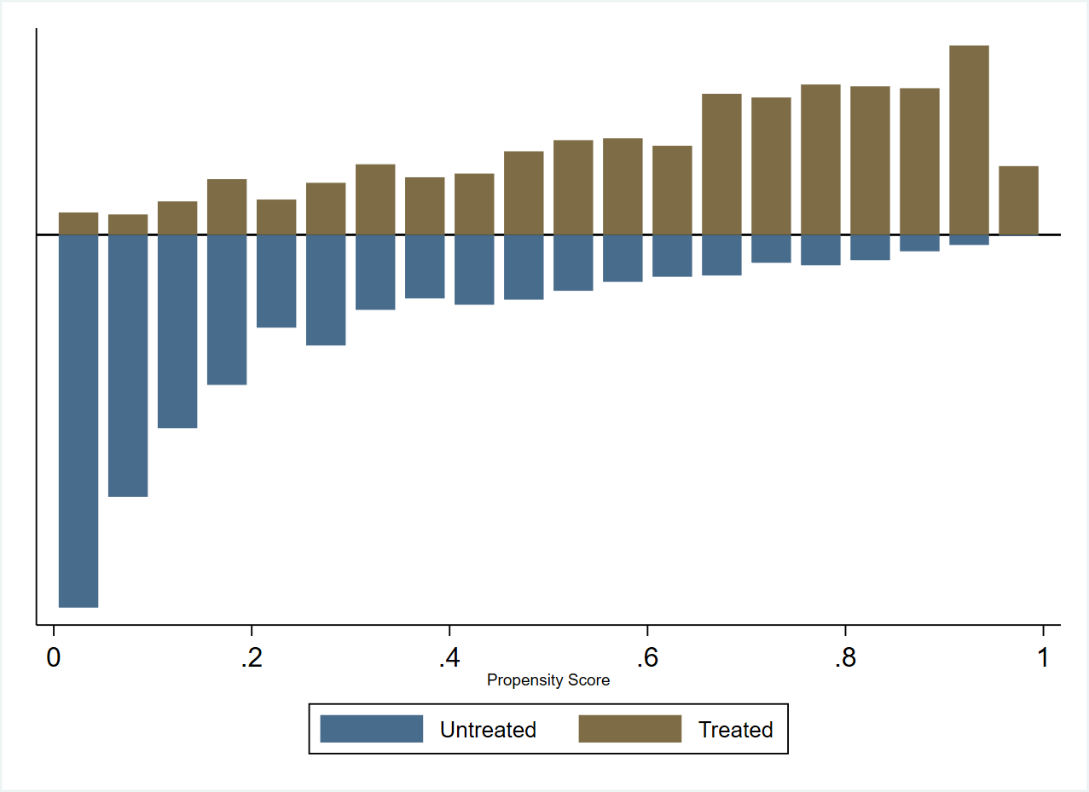

Supplement: S1 Fig — (DOCX) [file pone.0294248.s001.docx]
