# Supplementary material for: Parental co-residence and young adults’ mental health
Source: PLoS One. 2023 Nov 29;18(11):e0294248. doi: 10.1371/journal.pone.0294248 (PMC10686488; doi:10.1371/journal.pone.0294248)
Supplement: S1 Table — (DOCX) [file pone.0294248.s002.docx]

| **Variable name** | **Description** | **Coding** |
| --- | --- | --- |
| Age | Age last birthday at date of interview | Categorical |
| Sex | Sex | 0 “Male” 1 “Female” |
| Income | Individual financial year gross wages & salary | Categorical |
| Education | Highest education level achieved | 1 "School" 2 "College / Other" 3 "University" |
| Labour force status | Current labour force status | 0 “Not in labour force” 1 “In labour force” |
| First Nations origin | Aboriginal or Torres Strait Islander origin | 0 “No” 1 “Yes” |
| Parents income | Annual gross household income – Mothers. If Mothers is missing use Fathers | Quintiles |
| Parents education | One or both parents undertook university education | 0 "No" 1 " Yes" |
| Parents remoteness | Remoteness area – Mothers. If Mothers is missing use Fathers | 0 "Not Major City" 1 "Major City" |
| Parents tenure | Tenure – Mothers. If Mothers is missing use Fathers. | 1 "Owner" 2 "Renter - Private" 3 "Renter - Government" |
| Parents dwelling type | Recorded dwelling type – Mothers. If Mothers is missing use Fathers. | 0 "Flat" 1 "Semi-detached" 2 "Detached" |
