# Supplementary material for: Parental co-residence and young adults’ mental health
Source: PLoS One. 2023 Nov 29;18(11):e0294248. doi: 10.1371/journal.pone.0294248 (PMC10686488; doi:10.1371/journal.pone.0294248)
Supplement: S2 Table — (DOCX) [file pone.0294248.s003.docx]

|  | **Pseudo-R2** | **P>chi2** | **Mean bias** | **Rubin’s B** | **Rubin’s R** |
| --- | --- | --- | --- | --- | --- |
| **Unmatched** | 0.324 | 0.000 | 35.3 | 153.1 | 0.68 |
| **Matched** | 0.005 | 0.273 | 3.5 | 16.8 | 1.21 |
