# Supplementary material for: Parental co-residence and young adults’ mental health
Source: PLoS One. 2023 Nov 29;18(11):e0294248. doi: 10.1371/journal.pone.0294248 (PMC10686488; doi:10.1371/journal.pone.0294248)
Supplement: S3 Table — *Data on parents of young adults was different in earlier waves because this first waves of data collection did not include information on parents not living with the child in years prior to the data collection. For this reason, data from this wave has not been used to compare changes in prevalence over time. (DOCX) [file pone.0294248.s004.docx]

|  | **2002** | | | | **2010** | | | | **2018** | | | |
| --- | --- | --- | --- | --- | --- | --- | --- | --- | --- | --- | --- | --- |
|  | **Co-resident (n=1384)** | | **Independent**  **(n=3938)** | | **Co-resident (n=1384)** | | **Independent**  **(n=3938)** | | **Co-resident (n=1384)** | | **Independent**  **(n=3938)** | |
|  | **%** | **95% CI** | **%** | **95% CI** | **%** | **95% CI** | **%** | **95% CI** | **%** | **95% CI** | **%** | **95% CI** |
| **Parent(s) married or living with partner*** |  |  |  |  |  |  |  |  | 89 | (86.69-91.12) | 87 | (84.36-89.04) |
| **Parent’s highest income (mean) $** | 49,000 | (45,269-52,732) | 46,445 | (39,480-53,409) | 69,700 | (66,108-73,296) | 61,684 | (58,284-65,083) | 93,709.00 | (88,841-98,579) | 81,003 | (76,178-85,827) |
| **Parent’s highest house value (mean) $** | 385,185 | (358,660-411,693) | 326,700 | (275,967-377,432) | 691,668 | (650,651-732,685) | 658,572 | (616,627-700,517) | 1,011,683 | (95,791-106,545) | 805,916 | (77,150-84,033) |
| **Parent’s housing cost quote (% of income)** | 16 | (13.93-17.33) | 18 | (15.02-21.58) | 16 | (15.30-17.32) | 21 | (17.93-24.54) | 19 | (17.21-21.32) | 20 | (18.61-22.10) |
| **Parent’s tenure** | | | | | | | | | | | | |
| *Homeowner* | 84 | (80.47-86.98) | 84 | (74.25-90.00) | 83 | (80.60-85.93) | 81 | (77.09-83,67) | 79 | (76.40-82.20) | 80 | (77.18-81.82) |
| *Renter - Private* | 9 | (7.12-11.66) | 11 | (6.69-18.19) | 12.06 | (9.98-12.50) | 14 | (11.75-17.51) | 17 | (14.70-20.09) | 16 | (14.35-18.44) |
| *Renter - Government* | 7 | (4.70-9.94) | 5 | (1.56-16.00) | 5 | (3.21-6.30) | 5 | (3.38-7.40) | 3 | (2.29-4.80) | 4 | (2.97-5.65) |
| **Parents dwelling type** |  |  |  |  |  |  |  |  |  |  |  |  |
| *Flat* | 2 | (1.39-3.53) | 3 | (1.09-6.62) | 4 | (2.96-6.04) | 7 | (4.87-9.45) | 4 | (2.77-5.53) | 8 | (5.80-10.62) |
| *Semi-detached* | 5 | (3.21-6.52) | 2 | (0.77-719) | 4 | (2.46-5.05) | 4 | (3.05-6.03) | 5 | (3.26-6.62) | 5 | (3.41-6.14) |
| *Detached* | 93 | (91.01-94.87) | 95 | (91.01-94.87) | 92 | (90.04-93.97) | 89 | (86.00-91.25) | 91 | (89.07-93.30) | 87 | (84.64-89.74) |
| **Parent’s location** | | | | | | | | | | | | |
| *Major city* | 78 | (74.72-81.44) | 68 | (59.21-75.80) | 79 | (75.57-81.27) | 61 | (57.30-64.53) | 77 | (74.38-79.73) | 55 | (51.98-57.74) |
| *Outside of major city* | 22 | (18.56-25.28) | 32 | (24.20-40.80) | 21 | (18.73-24.43) | 39 | (35.47-42.42.70) | 23 | (20.27-25.62) | 45 | (42.26-48.02) |
